# Integrated NGS and ddPCR analysis of RAS/RAF mutations in metastatic colorectal cancer among Kazakhstani patients: a pilot study

**DOI:** 10.3389/fmed.2026.1884372

**Published:** 2026-08-11

**Authors:** Gulmira Kulmambetova, Pavel Tarlykov, Dana Auganova, Alua Gusmaulemova, Botakoz Kurentay, Meiram Mamlin

**Affiliations:** 1 Department of Genomics, National Center for Biotechnology, Astana, Kazakhstan; 2 Multidisciplinary Surgery Center, National Research Oncology Center, Astana, Kazakhstan

**Keywords:** BRAF, colorectal cancer, ddPCR, Kazakhstan, KRAS, mutation

## Abstract

**Background:**

Colorectal cancer (CRC) is one of the leading causes of cancer-related morbidity and mortality worldwide, and metastatic colorectal cancer (mCRC) is associated with poor prognosis and limited treatment options. Mutations in the *KRAS, BRAF* oncogenes are key molecular drivers of colorectal cancer development and promote activation of the MAPK/ERK signaling pathway. The presence of mutations serves as an important prognostic biomarker for response to targeted therapy against EGFR. However, molecular epidemiological data on RAS/RAF mutations in patients with colorectal cancer from Kazakhstan remain limited. Therefore, the aim of this study was to characterize the *KRAS*, *BRAF* mutation profile in patients with metastatic colorectal cancer from Kazakhstan using targeted NGS and ddPCR approaches.

**Methods:**

Formalin-fixed, paraffin-embedded (FFPE) tumor tissue samples from 30 patients with metastatic colorectal cancer (mCRC), including samples from primary tumors and liver metastases, were analyzed. Genomic DNA was isolated and analyzed for mutation hotspots in the *KRAS* and *BRAF* genes using droplet digital PCR (ddPCR). Mutation profiles of the *KRAS*, *BRAF* genes were also confirmed using targeted next-generation sequencing.

**Results:**

*KRAS* mutations were detected in 55.6% of primary tumors and 44.4% of metastatic samples by ddPCR, while *BRAF* mutations were present in 7.4 and 16.7% of cases, respectively. Targeted sequencing identified *KRAS* mutations in 65.2% of tumors and 64.3% of metastases, *BRAF* mutations in 4.4 and 14.3% of samples. The most frequent alterations were observed in codons 12, 13 of the *KRAS* gene. Comparative analysis of tumor-metastasis pairs revealed both common driver mutations and site-specific variants, indicating potential molecular heterogeneity.

**Conclusion:**

*KRAS* mutations are the most common genetic alterations in metastatic colorectal cancer, while *BRAF* mutations are less common. The combination of targeted next-generation sequencing and droplet digital PCR improves the accuracy of mutation detection in FFPE samples. These data clarify the molecular characteristics of metastatic colorectal cancer in Kazakhstan and support the development of precision oncology approaches in clinical practice.

## 1 Introduction

Colorectal cancer (CRC) remains one of the leading causes of cancer-related morbidity and mortality worldwide. According to global cancer statistics, CRC is among the three most frequently diagnosed malignancies and represents a significant public health challenge (1). A significant proportion of patients are diagnosed at late stages or subsequently develop metastatic colorectal cancer (mCRC), which is associated with a significantly worse prognosis and limited treatment options (2). In Kazakhstan, the incidence of CRC has been steadily increasing in recent decades, making it one of the most common oncological diseases in the country and highlighting the urgent need to improve diagnostic and prognostic strategies, particularly for metastatic disease (3, 4).

The development of colorectal cancer is a complex process caused by the accumulation of genetic and epigenetic changes that disrupt key signaling pathways regulating cell growth, differentiation, and apoptosis. Among these pathways, the mitogen-activated protein kinase (MAPK)/extracellular signal-regulated kinase (ERK) cascade plays a central role in colorectal carcinogenesis (5). *KRAS* and *BRAF* play a key role in this signaling pathway, acting as molecular switches that transmit signals from activated epidermal growth factor receptors (EGFR) to downstream effector mechanisms that regulate cell proliferation and survival *(**6**)*. *KRAS* encodes a small GTPase of the RAS family that functions as a molecular switch between GTP-active and GDP-inactive forms. Activating mutations, predominantly localized in codons 12 and 13 of exon 2, as well as codons 61 and 146, lead to sustained activation of the MAPK signaling pathway, independent of EGFR stimulation. As a result, cell proliferation is enhanced, apoptosis is suppressed, and tumor progression and metastasis are accelerated. *KRAS* mutations are detected in approximately 40–50% of colorectal cancer cases and are considered early molecular events in carcinogenesis (7, 8).

*BRAF*, located downstream of *KRAS*, encodes a serine/threonine kinase that mediates the activation of MEK and ERK in the MAPK cascade. The most common mutation in colorectal cancer is V600E, which leads to persistent kinase activation and enhanced MAPK signaling. Although *BRAF* mutations are found in approximately 8–12% of patients with colorectal cancer, they are strongly associated with unfavorable clinicopathological characteristics, including low tumor grade, right-sided localization, high metastatic potential, and worse prognosis (9, 10).

Despite the clinical significance of *KRAS* and *BRAF* mutations in metastatic colorectal cancer (mCRC), data on their prevalence and molecular characterization in populations of Central Asia, including Kazakhstan, remain limited. One of the few regional studies described the frequency of *KRAS* mutations in colorectal cancer patients from Kazakhstan, but it did not specifically address metastatic disease or comprehensive *BRAF* profiling (11, 12). Most of the available data on mutation rates and testing strategies come from studies in larger Asian and international cohorts (13), however, data for Kazakhstan remain scarce and are often limited to cases of colorectal cancer in general, rather than mCRC. The limited population-specific molecular data highlights the need to apply robust mutation profiling methods to available tumor samples to more accurately characterize the genomic landscape of metastatic colorectal cancer in this underrepresented population.

Accurate detection of RAS/RAF mutations is clinically important in metastatic colorectal cancer, as these alterations are key predictive biomarkers of response to anti-EGFR therapy. The European Society for Medical Oncology (ESMO) and the National Comprehensive Cancer Network (NCCN) recommend comprehensive testing for RAS (*KRAS* and *NRAS*) and *BRAF* mutations before treatment. Activating RAS mutations predict failure of anti-EGFR therapy, making accurate mutation detection essential for appropriate treatment selection. In this regard, comprehensive molecular profiling has become an integral part of precision oncology. However, traditional PCR methods used to detect hotspot mutations are limited in their capabilities for multigene analysis and detection of rare variants.

Modern advances in molecular diagnostics have facilitated the introduction of ddPCR as a highly sensitive quantitative approach for detecting clinically significant *KRAS* and *BRAF* mutations in various colorectal cancer samples (14, 15). Although ddPCR has been extensively validated for the analysis of circulating tumor DNA in liquid biopsy settings, demonstrating high concordance with tissue genotyping and improved sensitivity to low-frequency variants (14, 16, 17), its technical advantages are also relevant for formalin-fixed paraffin-embedded (FFPE) tumor tissues (18). Due to its tolerance for fragmented DNA and the ability to provide absolute quantification of mutant alleles, ddPCR enables reliable mutation profiling in formalin-fixed paraffin-embedded (FFPE) samples, which are the most readily available material in routine pathology practice (15, 18).

Additionally, targeted next-generation sequencing (NGS) has emerged as a powerful method for comprehensive mutation profiling in cancer (19–21). Targeted sequencing panels enable the simultaneous analysis of multiple oncogenes and hotspots with high sensitivity and specificity using a relatively small amount of DNA. This approach is particularly important for the analysis of formalin-fixed paraffin-embedded (FFPE) tumor samples, which represent the most common source of clinical material in routine pathology laboratories. By simultaneously detecting mutations in genes such as *KRAS*, *BRAF*, targeted sequencing provides a comprehensive overview of the molecular landscape of colorectal tumors and enables the identification of both common driver mutations and less common variants.

Thus, the integration of targeted sequencing with highly sensitive methods such as ddPCR represents a robust mutation profiling strategy in clinical oncology.

In this study, we performed mutation profiling in FFPE tumor tissue samples from patients with metastatic colorectal cancer to characterize mutations in RAS/RAF pathway genes using a combined approach including targeted next-generation sequencing and droplet digital PCR.

## 2 Materials and methods

### 2.1 Patients and tumor characteristics

Formalin-fixed, paraffin-embedded (FFPE) tissue sections of primary tumor and liver metastases were obtained from a heterogeneous samples of 30 patients with mCRC. The histopathological diagnosis was conducted by the experienced pathologists. Histological typing, grading, localization and staging of tumors were determined using the recommendation according to WHO and IUCC.^1^ The main criterion of patients included in the study was the presence of at least one metastatic.

### 2.2 DNA isolation

Genomic DNA from FFPE tissue was isolated with the commercial kit QIAamp DNA FFPE Advanced Kit (Qiagen, Germany). Briefly, FFPE sections were lysed and shacked 1 h at 65 °C and 1 h at 90 °C, respectively. DNA was bound to the spin filter, washed and eluted to 40 μL of elution solution. The concentration of DNA was measured by Qubit Fluorometer 2.0 (Invitrogene, California, USA) with Qubit DNA BR Assay Kit (Life Technologies, California, USA).

### 2.3 Droplet digital PCR for *KRAS/BRAF* mutations screening

ddPCR™ *KRAS* G12/G13 Screening Multiplex Kit 1,863,506, and ddPCR *BRAF* V600 Screening Kit 12,001,037 (Bio-Rad Laboratories, Hercules, California, USA) for 7 *KRAS* mutations (G12A, G12C, G12D, G12 V, G12R, G12S, G13D), which contains 20x concentrated assay mix and two tubes of 2x concentrated ddPCR supermix for probes, was used. The total volume of 20 μL ddPCR Master mix was prepared with 10 μL of 2x supermix for probes, 2 μL of assay mix, ultrapure water and 100 ng of DNA in variable volume. Each PCR reaction mix was loaded into the sample well of DG8™ cartridge (Bio-Rad Laboratories, Hercules, California, USA) and 70 μL of Droplet Generation Oil for Probes (Bio-Rad Laboratories, Hercules, California, USA) was added into each oil well of cartridge. The full cartridge was placed into QX200™ Droplet Generator (Bio-Rad Laboratories, Hercules, California, USA), which separated PCR mix into ~ 20,000 droplets in water-in-oil emulsion. Emulsified PCR mixes were then transferred to the 96-well plate by multichannel pipette, the plate was heat sealed with foil in PX1 Plate Sealer (Bio-Rad Laboratories, Hercules, California, USA) and placed into the T100™ Thermal Cycler (Bio-Rad Laboratories, Hercules, California, USA). The thermal cycling conditions of PCR reactions with ramp set up as ~ 2 °C/s were 10 min at 95 °C and 40 cycles with 30 s denaturation at 94 °C and 1 min annealing extension at 55 °C. The enzyme inactivation was run 10 min at 98 °C. The samples were held at 4 °C with cooling ramp set up as ~ 1 °C/s. After amplification, the 96-well plate was loaded to the QX200™ Droplet Reader (Bio-Rad Laboratories, Hercules, California, USA) and droplets from each well were analyzed using QuantaSoft software (Bio-Rad Laboratories, Hercules, California, USA). Four main populations of droplets were shown by the analysis of emission in HEX or FAM wavelenghts—empty droplets without DNA, droplets containing only wild-type DNA with high HEX amplitude, droplets containing only mutant DNA with high FAM amplitude and droplets with both type of DNA with high HEX and also FAM amplitude.

### 2.4 Targeted sequencing

Targeted next-generation sequencing of *KRAS/NRAS* and *BRAF* was performed using the fastGEN Solid Cancer Kit II RUO (BioVendor, Czech Republic), according to the manufacturer’s instructions.^2^ The fastGEN Solid Cancer Kit II RUO covered a broader range of KRAS mutations, including variants in exons 2, 3, 4A, and 4B. Amplicon libraries were prepared from tissue-extracted genomic DNA (FFPE) to detect somatic mutations in the *KRAS* and *BRAF* genes. Following PCR amplification, the libraries were indexed, purified, and quantified prior to sequencing. Sequencing was performed on an Illumina-compatible platform using paired-end reads.

### 2.5 Data analysis

Data obtained from QX200™ Droplet Reader were processed and analyzed by commercial QuantaSoft v.1.7 Software (Bio-Rad Laboratories, Hercules, California, USA). The threshold was set up using normal genomic DNA with wt*KRAS* and the commercial DNA from LS174T with mutated *KRAS* (G12D) and the cutoff for fractional abundance (FA) was calculated. The samples with FA ≥ 0.5 were assessed as positive, and samples with FA < 0.5 were assessed as negative.

Statistical analysis was performed using R. Studio version 4.2.0 software. Fisher’s exact test was used to assess differences in the frequency of *KRAS* and *BRAF* mutations between primary tumors and metastatic sites, even with a small sample size. Statistical significance was defined as *p* < 0.05.

## 3 Results

### 3.1 Patient and tumor characteristics

The study included 30 patients with metastatic colorectal cancer (mCRC). The median age was 64 years (range: 44–87), with an equal gender distribution (50% men, 50% women). The ethnic composition included 50% Asian and 50% Caucasian. The tumor localization was predominantly colon (60%), followed by rectum (33.3%). All tumors were classified as moderately (G2) or poorly differentiated (G3); well-differentiated tumors were not observed (Table 1).

**Table 1:** Clinicopathological characteristics of 30 patients with metastatic colorectal cancer.

Characteristic	Value
Age, years	
Mean	64
Range	44–87
Gender, n (%)	
Male	15 (50)
Female	15 (50)
Ethnicity, n (%)	
Asians	15 (50)
Europeans	15 (50)
Tumor site, n (%)	
Colon	18 (60)
Rectum	10 (33.3)
Unknown	2 (6.7)
Tumor differentiation, n (%)	
Moderate	16 (53.3)
Poor	12 (40)
Unknown	2 (6.7)
Tumor, n (%)	27 (90)
Metastasis, n (%)	18 (60)

### 3.2 *KRAS*, *BRAF* mutation spectrum

Mutational analysis of the *KRAS, BRAF,* genes was performed in primary tumor samples (*n* = 19) and metastatic samples (*n* = 13).

All detected pathogenic variants passed quality filters (FILTER = good) and were confirmed by high sequencing depth (701–9,195×). Variant allele frequencies ranged from 6.21 to 38.83%. Most variants demonstrated high genotypic quality (GQ = 255), indicating high confidence in variant detection.

Even low-frequency variants (VAF ~ 6.21%) were confirmed by at least 20 mutant reads, suggesting that they represent true somatic variants and not sequencing artifacts. Low-frequency variants (VAF ~ 2–6%) were excluded from the analysis. Therefore, the sequencing analysis covered 20 patients, from whom 37 samples were tested, with somatic mutations detected in 27 samples.

The detected alterations were mainly located in known hotspot codons 12, 13, and 61 of RAS genes and codon 600 of *BRAF* (Table 2).

**Table 2:** Types of *KRAS, BRAF* mutations.

Gene	HGVSc	HGVSp	Mutation	Tumor (*n* = 16)	Metastasis (*n* = 11)
*KRAS*	c.35G > T	p. Gly12Val	G12V	2 (12.5)	2 (18.18)
	c.38G > T	p. Gly13Val	G13V	12 (75)	7 (63.64)
	c.181C > A	p. Gln61Lys	Q61K	1 (6.25)	0
*BRAF*	c.1799 T > A	p. Val600Glu	V600E	1 (6.25)	2 (18.18)

Amino acids: E, Glutamic acid; G, Glycine; K, Lysine; Q, Glutamine; V, Valine. KRAS, Kirsten rat sarcoma viral oncogene homolog; BRAF, v-Raf murine sarcoma viral oncogene homolog B.

The number of reads supporting the alternative allele ranged from 127 to 2,254, which significantly exceeds the minimum thresholds recommended for reliable identification of somatic mutations. The frequency of the variant allele ranged from 7.87 to 38.83%, which corresponds to reliably detectable somatic mutations. The pathogenic *KRAS* p. Gly13Val mutation was detected most frequently, while *KRAS* p. Gly12Val and *BRAF* p. Val600Glu were significantly less common. In *KRAS*, the most frequent mutations were p. Gly13Val (G13V), detected in 75% of primary tumors and 63.64% of metastases, respectively. Other variants included G12V (12.5% of tumors; 18.18% of metastases), and Q61K (6.25% of tumors only).

In the *BRAF* gene, the mutation was p. Val600Glu (V600E), found in 6.25% of tumors and 18.18% of metastases. V600E mutation was detected with less frequency.

**Figure 1:**
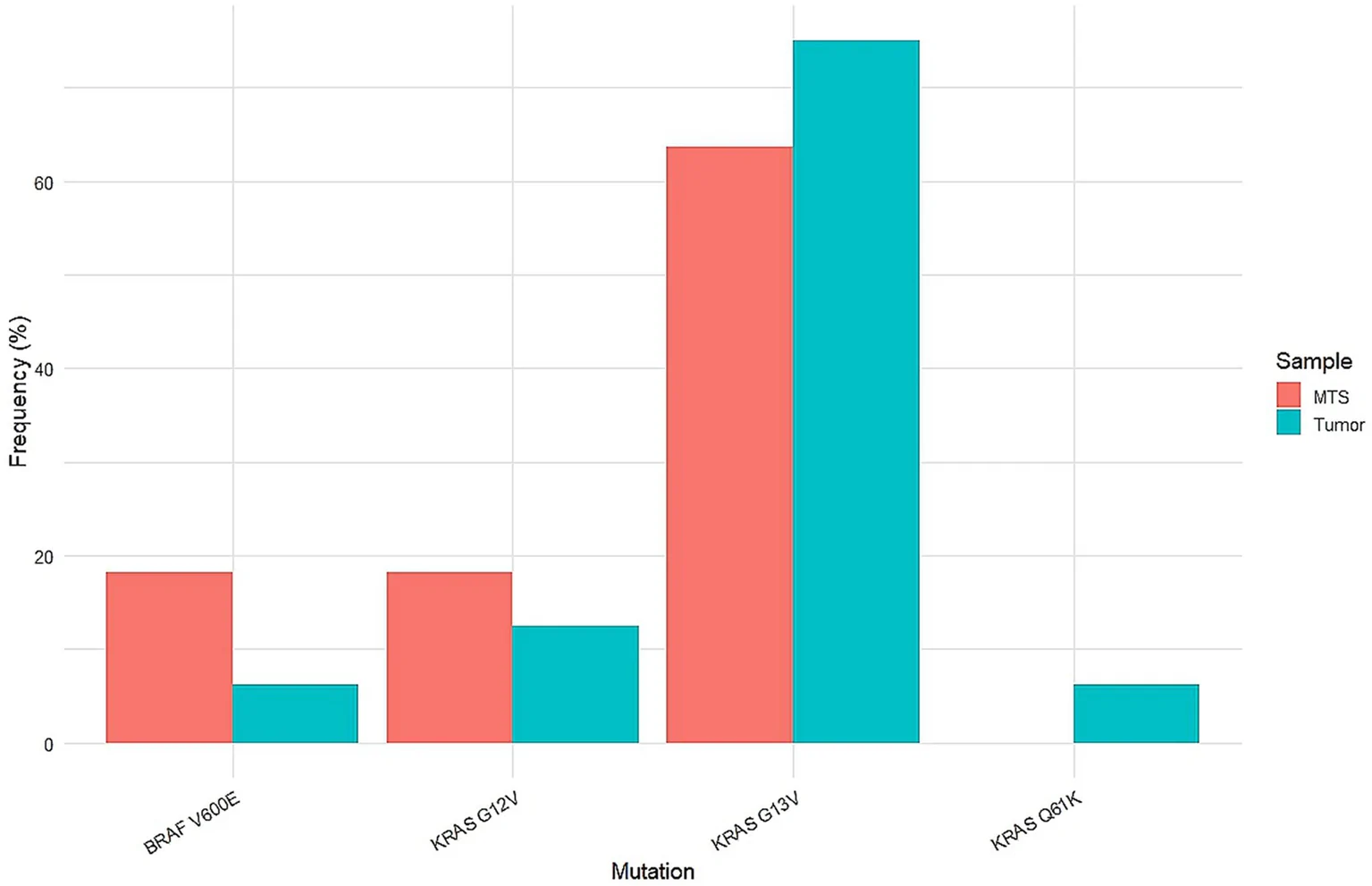
Frequency of *KRAS, BRAF* mutations in tumors and metastases.

Analysis of *KRAS* and *BRAF* mutation rates demonstrated a higher overall prevalence of *KRAS* alterations compared to *BRAF* in both primary tumors and metastases (Figure 1). The high frequency of *KRAS* mutations in the primary tumor and metastases may be related to the small sample size and rarity of the mutations.

### 3.3 Genes mutation status in primary tumors and metastases

Using ddPCR, *KRAS* and *BRAF* mutation status was assessed in primary tumor and metastasis samples from mCRC patients (Table 3).

**Table 3:** Distribution of the *KRAS*/*BRAF* status among mCRC patients by ddPCR.

Gene	Mutation status	Tumor *n* = 27 (%)	Metastasis *n* = 18 (%)	*p*-value, 95% CI
*KRAS*	Mutant	15 (55.6)	8 (44.4)	0.55 (0.40–6.15)
	Wild type	12 (44.4)	10 (55.6)	
*BRAF*	Mutant	2 (7.4)	3 (16.7)	0.37 (0.03–3.99)
	Wild type	25 (92.6)	15 (83.3)	

*KRAS* mutations were detected in 55.6% (15 of 27) of primary tumors and 44.4% (8 of 18) of metastatic samples, while the wild-type genotype was observed in 44.4 and 55.6% of cases, respectively. The difference between tumor and metastasis groups was not statistically significant (*p* = 0.55; 95% CI: 0.40–6.15).

*BRAF* mutations were detected in 7.4% (2 of 27) of primary tumor samples and 16.7% (3 of 18) of metastatic samples, while the wild-type genotype was observed in 92.6 and 83.3% of cases, respectively. No statistically significant differences were found between groups (*p* = 0.37; 95% CI: 0.03–3.99).

Overall, ddPCR analysis did not reveal significant differences in the distribution of *KRAS* or *BRAF* mutation status between primary tumors and metastatic lesions (Table 3).

Targeted sequencing revealed mutations in *KRAS*, *BRAF* in both tumor (*n* = 23) and metastatic samples (*n* = 14; Table 4).

**Table 4:** Distribution of *KRAS/BRAF* mutations in tumors and metastases by targeted sequencing.

Gene	Mutation status	Tumor *n* = 23 (%)	Metastasis *n* = 14 (%)	CI 95%	*p*-value
*KRAS*	Mutant	15 (65.2)	9 (64.3)	0.20–5.07	1
	Wild type	8 (34.8)	5 (35.7)		
*BRAF*	Mutant	1 (4.4)	2 (14.3)	0.004–5.95	1
	Wild type	22 (95.7)	12 (85.7)		

*KRAS* mutations were detected in 65.2% of tumors and 64.3% of metastases (*p* = 1; 95% CI: 0.20–5.07). *BRAF* mutations were found in 4.4% of tumors and 14.3% of metastases (*p* = 1; 95% CI: 0.004–5.95).

The lack of statistically significant findings may reflect insufficient statistical power rather than the absence of underlying biological differences. It should also be noted that the ddPCR kit (*KRAS* G12/G13 Screening Multiplex Kit) targets a limited panel of seven specific mutations, while NGS allows for the detection of a wider range of variants.

### 3.4 Concordance between primary tumors and metastatic sites

A comparative analysis of somatic mutations between primary colon tumors and corresponding metastatic lesions revealed both common and lesion-specific alterations in the *KRAS*, *BRAF* genes. Detailed data on the mutation spectrum in paired samples are presented in Table 5.

**Table 5:** Types of *KRAS, BRAF* mutations in 2 matched samples.

Patient Id	Gene	HGVSc	HGVSp	Exon	Primary tumor VAF (%)	Metastasis VAF (%)	Status
P1	*KRAS*	c.38G > T	p. Gly13Val	2/5	23.41	9.65	Shared
P6	*KRAS*	c.38G > T	p. Gly13Val	2/5	23.28	8.31	Shared
P6	*KRAS*	c.181C > A	p. Gln61Lys	3/5	7.87		Primary only
P6	*KRAS*	c.35G > T	p. Gly12Val	2/5		27.92	Mts only

Matching mutations in the primary tumors and metastases were predominantly found in the *KRAS* gene, including the p. Gly13Val variants identified in patients P1, P6 with varying levels of variant allele frequency (VAF). However, a number of alterations were restricted exclusively to primary tumors, including *KRAS* p. Gln61Lys.

Additionally, mutations specific to metastatic lesions were identified, including *KRAS* p. Gly12Val. In some cases, metastatic samples demonstrated a higher variant allelic frequency for example, *KRAS* p. Gly12Val in patient P6. Although only a small number of paired samples were available for analysis, the observed discordance in mutation profiles between primary tumors and matched metastases provides preliminary evidence of molecular heterogeneity. Further studies with larger cohorts are required to determine the prevalence and clinical significance of these findings.

The presence of multiple KRAS hotspot mutations (codons 12, 13, and 61) in the same patient is rare and highlights significant heterogeneity. This heterogeneity may have implications for molecular diagnostics, therapeutic decision-making, and resistance mechanisms.

Overall, the results indicate partial overlap in the mutational profile between the primary tumors and metastases, with the presence of common driver mutations and lesion-specific variants, reflecting the pronounced molecular heterogeneity (clonal evolution) of colorectal cancer during disease progression.

### 3.5 Association of *KRAS* mutations with clinicopathological characteristics

The relationship between *KRAS* mutation status (determined by ddPCR) and clinicopathological characteristics was analyzed in 27 patients with colorectal cancer (Table 6). *KRAS* mutations were detected in 15 patients (55.6%), while 12 (44.4%) were wild-type. *Post hoc* power analysis revealed that with sample sizes of 12 and 15, the study had 51.0% power to detect a large effect size (Cohen’s d = 0.8) at a two-sided significance level of 0.05. This value is significantly below the recommended threshold of 80%, indicating insufficient power. Therefore, the limited sample size may have reduced the ability to detect true associations.

**Table 6:** Associations between *KRAS* mutation and clinicopathological characteristics ddPCR.

Characteristics	All (*n* = 27)	*KRAS* wild-type (*n* = 12)	*KRAS* mutant (*n* = 15)	*p*-value
Age (mean ± SD), years^a^	63.2 ± 10.9	63 ± 8.0	63.3 ± 13.1	0.94
Gender, n (%)^b^				
Male	14	8 (57.1)	6 (42.9)	0.25
Female	13	4 (30.8)	9 (69.2)	
Ethnicity, n (%)^b^				
Asians	12	4 (33.3)	8 (66.7)	0.44
Europeans	15	8 (53.3)	7 (46.7)	
Tumor site, n (%)^b^				
Right	4	3 (75)	1 (25)	0.29
Left	23	9 (39.1)	14 (60.9)	
Tumor differentiation, n(%)^b^				
Moderate	14	4 (28.6)	10 (71.4)	0.12
Poor	11	7 (63.6)	4 (36.4)	
NA	2	1	1	
Histological type, n (%)^b^				
Mucinous	4	3 (75)	1 (25)	0.31
Non-mucinous	17	7 (41.2)	10 (58.8)	
NA	6	2	4	

a Independent t-test.

b Fisher’s exact test.

SD, standard deviation.

The mean patient age did not differ between groups (63.2 ± 10.9 vs. 63 ± 8.0 years; *p* = 0.94). No statistically significant associations were found between *KRAS* mutation status and gender (*p* = 0.25), ethnicity (*p* = 0.44), tumor location (*p* = 0.29), grade of differentiation (*p* = 0.12), or histologic type (*p* = 0.31).

Overall, KRAS mutation status did not demonstrate statistically significant associations with the clinicopathological parameters evaluated in this patient cohort. These negative findings may reflect insufficient statistical power rather than the absence of underlying biological differences.

Mutation status of *KRAS* was analyzed in 27 colorectal cancer patients (Supplementary Table 1). Overall, *KRAS* mutations were detected in 55.6% (15/27) of cases, while 44.4% (12/27) showed no detected mutations (wild type).

Regarding tumor localization, *KRAS* mutations were most frequent in tumors of the sigmoid colon and descending colon (66.7% each), followed by the rectosigmoid flexure and rectum (50% each). *KRAS* mutations were also identified in the single cases of the transverse colon and splenic flexure (100% each), whereas no mutations were detected in tumors of the cecum (n = 2) or ascending colon (n = 1). Overall, the majority of tumors with detected mutations were located in the left colon and rectum (Supplementary Table 1).

### 3.6 Relationship between *BRAF* mutations and tumor localization

The relationship between *BRAF* mutation status and tumor location was analyzed in 27 patients with colorectal cancer (Supplementary Table 2). The majority of tumors were located in the left colon (85.2%), while 14.8% were located on the right side.

Wild-type *BRAF* was detected in all right-sided colon tumors. Conversely, among left-sided tumors, *BRAF* mutations were detected in 8.7% (2/23) of cases, while the wild-type gene was detected in 91.3% (21/23).

No statistically significant association was found between *BRAF* mutation status and tumor location (Fisher’s exact test, *p* = 1).

## 4 Discussion

This study evaluated the spectrum and distribution of *KRAS, BRAF* mutations in primary tumors and metastatic lesions of patients with metastatic colorectal cancer. The identified mutations were predominantly located in known hot spots of the RAS–RAF signaling pathway, including codons 12, 13, and 61 of the *KRAS* gene, as well as codons 600 of the *BRAF* gene, which are recognized drivers of colorectal tumorigenesis.

*KRAS* mutations were the most frequently detected genetic alterations, consistent with previous studies that implicate *KRAS* as a leading oncogenic driver in colorectal cancer (7, 8). In the study cohort, G13V, G12V, and Q61K mutations were predominant, suggesting a key role for these mutational hotspots in tumor progression and the development of resistance to anti-EGFR therapy.

*BRAF* mutations were detected significantly less frequently, which is also consistent with global epidemiological data, which indicate that *BRAF* alterations are found only in a relatively small subset of colorectal cancer patients (9, 10).

Comparative analysis of primary tumors and metastatic lesions revealed partial mutation overlap: along with several common changes, tumor-specific and metastasis-specific variants were also detected. These results indicate pronounced molecular heterogeneity and clonal evolution of the tumor during metastatic progression, in which subclonal populations can either expand or form anew at metastatic sites. In some cases, a higher variant allele frequencies in metastatic lesions further indicates selective clonal expansion during tumor dissemination. The observed discordance between paired primary tumors and metastatic lesions further supports the concept that clinically significant mutations can develop during disease progression. Therefore, relying solely on the molecular profile of the primary tumor may not be sufficient to fully reflect the genomic landscape of metastatic disease in all patients (22).

Metastatic colorectal cancer is characterized by significant genomic heterogeneity. Differences in the molecular profiles of tumors on the right and left sides suggest distinct carcinogenesis pathways, which may determine differences in the clinical course of the disease and treatment outcomes (23).

Metastatic colorectal cancer remains a molecularly heterogeneous disease associated with adverse clinical outcomes. Mutations in *KRAS* and *BRAF* are among the most important molecular alterations in this cancer and are associated with limited response to anti-EGFR therapy and unfavorable clinical outcomes (24). Therefore, identification of these mutations is essential for appropriate patient stratification and treatment selection. Continued research on the biological mechanisms underlying these mutations and the development of personalized therapeutic strategies may improve treatment response and survival in patients with metastatic colorectal cancer.

No statistically significant associations were found between *KRAS* mutation status and clinicopathological characteristics such as age, gender, grade of differentiation, or tumor location. These results reflect the limited sample size, which reduces the statistical power to detect existing biological associations. Similarly, *BRAF* mutations did not demonstrate a significant association with tumor location, although they were detected exclusively in left-sided tumors in this cohort. Therefore, these results should be considered preliminary. Validation in larger, independent cohorts is necessary to determine the presence of clinically significant associations in the Kazakhstani population.

Overall, the mutation distribution identified in this study is consistent with the range described in international populations; however, the observed differences in frequency may be due to geographic and population characteristics. Several studies conducted in Caucasian and Asian populations with сolorectal сancer have reported that *KRAS* mutations are not significantly associated with clinicopathological characteristics, including patient age, sex, ethnicity, tumor location, degree of differentiation, or mucinous status (25–27).

Evidence suggests that these mutations may be associated with patient sex and the anatomical location of the tumor (28). Several studies have reported inconsistent findings regarding the relationship between *KRAS* mutations and tumor location in colorectal cancer. Some studies report a higher frequency of *KRAS* mutations in right-sided colon tumors (29), while others have noted a higher prevalence in left-sided colon tumors (30). Specifically, mutations in codons 12 and 13 of the *KRAS* gene have been shown to be significantly more common in right-sided colon cancer (31, 32). Furthermore, a number of studies suggest that mutations in codon 13 of *KRAS* may be more common in women with rectal tumors (33).

The frequency of *KRAS* and *BRAF* gene mutations in the study cohort is generally consistent with previously published data on colorectal cancer, despite some variability between studies. In our analysis, *KRAS* mutations were detected in 55.6% of cases, a frequency slightly higher than the 18–52% range reported in most international populations. A comparative analysis of various studies demonstrates a relatively stable prevalence of these mutations among patients with colorectal cancer, with a frequency of approximately 44–49% in most cohorts, consistent with data from the global literature.

Several international studies have assessed the prevalence of *KRAS* and *BRAF* mutations in patients with colorectal cancer, including metastatic forms of the disease (mCRC).

In a study conducted in the United States, Jae Gong et al. analyzed 138 tumor tissue samples using comprehensive genomic profiling using NGS. The mutation frequency was 49.3% for *KRAS*, 2.2% for *NRAS*, and 6.5% for *BRAF* (34).

In an Italian cohort (*n* = 219) studied by Elisa Isnardi and colleagues, *KRAS* mutations were detected in approximately 45% of patients, while the frequency of *NRAS* and *BRAF* mutations was approximately 5 and 8%, respectively (35). Similar results were obtained in a large Turkish study (*n* = 694) by Ilker Gökmen et al., where the frequency of *KRAS* mutations (exons 2–4) was 47.8%, *NRAS* mutations 4.5%, and *BRAF* mutations 5.3% (36).

In a comparative analysis of Arab and Western populations by Humayd O. Al-Shamsi et al., *KRAS* mutations were detected in 44% of Arab patients and 52% of patients in the Western cohort, while *NRAS* and *BRAF* mutations were observed in approximately 4% of patients in the Arab group (37).

In another Italian study (Bruera et al., *n* = 63), the frequency of *KRAS* mutations reached 66.7%, while *NRAS* and *BRAF* mutations were detected in 16.4 and 7.5% of cases, respectively (38). In a study conducted in Saudi Arabia (Sahar Saharti), the frequency of *KRAS* mutations was 45.2%, while *NRAS* and *BRAF* mutations were found in 2.2% of patients (39). Unlike *KRAS*, *NRAS* mutations were significantly less common, typically not exceeding 2–5% of cases. However, a higher frequency of *NRAS* (16.4%) was observed in the study by Bruera et al., which may be due to the small sample size and inclusion of rare mutations (38).

*BRAF* mutations were detected in approximately 2–8% of patients, consistent with previously published data. The *BRAF* V600E mutation is known to be associated with a more aggressive disease course and poor prognosis in patients with metastatic colorectal cancer.

Despite differences between countries and study cohorts, the overall structure of the mutational spectrum remains similar: *KRAS* mutations predominate, while *BRAF* are significantly less common. These results confirm the key role of RAS/RAF mutation analysis in the selection of targeted therapy, especially when prescribing anti-EGFR drugs.

It is known that MSI and frequency of *KRAS* and *BRAF* mutations differed by demographics and also racial/ethnic disparities in overall survival differed by mutation (40).

Overall, the mutation frequencies observed in our cohort are comparable to those reported in other international populations, supporting the global pattern in which *KRAS* mutations are significantly more common than *BRAF* mutations in colorectal cancer. However, the identified regional differences highlight the need for population-specific molecular studies, which is particularly important for optimizing diagnostic testing and selecting targeted therapy in patients with colorectal cancer.

Our results are consistent with current understanding of the high clinical value of multimarker approaches in colorectal cancer (41). It has previously been shown that new prognostic biomarkers such as micronucleus frequency and telomerase activity can complement traditional methods for assessing disease progression (42). Our data also confirm that combining different types of biomarkers may be more clinically informative than using individual markers alone. Along with established molecular markers, including *KRAS* and *BRAF* mutations, integrating *Fusobacterium nucleatum* detection with inflammatory biomarkers may provide a more comprehensive characterization of tumor biology and its microenvironment (43). This comprehensive approach has the potential to improve patient stratification and develop personalized diagnostic, prognostic, and therapeutic strategies for colorectal cancer.

Several limitations should be considered when interpreting the obtained results. First, the relatively small sample size may have reduced the statistical power of the analysis and limited the detection of significant associations between mutation status and clinicopathological characteristics. Second, the presence of unmatched paired samples of primary tumors and metastases in some patients limits the ability to fully assess concordance and clonal evolution. Also, a limitation of the study is that ddPCR and targeted NGS were performed on different subsets of patient. Consequently, direct concordance analysis between the two methods could not be performed. Future studies should evaluate both techniques using the same set of tumor samples to assess analytical agreement. A limitation of this study is the exclusion of *NRAS* mutations from the final analysis, as the allele frequencies of all detected variants were below the predetermined quality threshold. Future studies with larger cohorts and improved analytical sensitivity are needed to assess the clinical significance of low-frequency *NRAS* mutations. Finally, the study focused on a limited gene panel (*KRAS, BRAF*), which means other potentially significant molecular alterations involved in colorectal cancer progression were not included in the analysis.

In conclusion, this study demonstrates that *KRAS* mutations are the most common genetic alterations in metastatic colorectal cancer, while *BRAF* mutations are significantly less common. The detected alterations are predominantly localized in known “hot spots” of the RAS–RAF signaling pathway. Comparative analysis of primary tumors and metastatic lesions revealed a partial overlap in mutational profiles, suggesting molecular heterogeneity and possible clonal evolution during disease progression. These data highlight the importance of molecular profiling of both primary and metastatic lesions for a deeper understanding of tumor evolution and the development of personalized therapeutic strategies for colorectal cancer.

## Data Availability

The data presented in the study are deposited in the NCBI Sequence Read Archive (SRA) repository, under BioProject accession number PRJNA1509116.

## Footnotes

1
https://www.uicc.org/what-we-do/sharing-knowledge/tnm

2
https://www.biovendor.com/fastgen-solid-cancer-kit-ii
