# Supplementary material for: Integrated NGS and ddPCR analysis of RAS/RAF mutations in metastatic colorectal cancer among Kazakhstani patients: a pilot study
Source: Front Med (Lausanne). 2026 Aug 11;13:1884372. doi: 10.3389/fmed.2026.1884372 (PMC13504644; doi:10.3389/fmed.2026.1884372)
Supplement: Supplementary file 1 [file Table_1.DOCX]

Supplementary Material

# 1 Supplementary Tables

Table 1. Relationship between occurrence of mutations in the *KRAS* gene and tumor localization in mCRC patients. *Lack of any examined mutations (wild type)

| Tumor location | Samples (n) | *Lack of mutations, n (%) | *KRAS* mutations, n (%) |
| --- | --- | --- | --- |
| All | 27 | 12 (44.4) | 15 (55.6) |
| Cecum | 2 | 2 (100) | 0 |
| Ascending colon | 1 | 1 (100) | 0 |
| Transverse colon | 1 | 0 | 1 (100) |
| Splenic flexure | 1 | 0 | 1 (100) |
| Descending colon | 3 | 1 (33.3) | 2 (66.7) |
| Sigmoid colon | 9 | 3 (33.3) | 6 (66.7) |
| Rectosigmoid flexure | 2 | 1 (50) | 1 (50) |
| Rectum | 8 | 4 (50) | 4 (50) |

Table 2. Relationship between occurrence of mutations in the *BRAF* gene and tumor localization in mCRC patients. *Lack of any examined mutations (wild type)

| Tumor location | Samples (n) % | *BRAF* gene status | Samples (n) % |
| --- | --- | --- | --- |
| Right side of large intestine | 4 (14.8) | Lack of mutations* | 0 |
|  |  | Presence of mutations | 0 |
| Left side of large intestine | 23 (85.2) | Lack of mutations* | 21 (91.3) |
|  |  | Presence of mutations | 2 (87) |
| P, Fisher | | 1 | |
